# Investigation of miRNAs Associated with Inflammation and Apoptosis in Patients with Idiopathic Trigeminal Neuralgia

**DOI:** 10.3390/diagnostics16060894

**Published:** 2026-03-18

**Authors:** Elif Simin Issı, Serap Tutgun Onrat, Hasibe Nesligül Gönen, Hakan Acar, Ülkü Türk Börü

**Affiliations:** 1 Department of Neurology, Afyonkarahisar Health Sciences University, Afyonkarahisar 03030, Türkiye; 2 Department of Medical Genetics, Afyonkarahisar Health Sciences University, Afyonkarahisar 03030, Türkiye; 3 Department of Neurology, Bursa Çekirge State Hospital, Bursa 16090, Türkiye; 4 Department of Neurology, Yalova University Faculty of Medicine, Yalova 77200, Türkiye

**Keywords:** trigeminal neuralgia, microRNA, apoptosis, inflammation, biomarker, ROC analysis, diagnostic accuracy

## Abstract

**Background**: Trigeminal neuralgia (TN) is a severe neuropathic pain disorder primarily diagnosed on clinical grounds, and objective molecular biomarkers that could support diagnosis remain limited. Increasing evidence suggests that inflammation–apoptosis interactions contribute to TN pathophysiology. **Methods**: In this exploratory prospective case–control study, circulating apoptosis-related microRNAs (miRNAs) were analyzed in 30 patients with idiopathic TN and 20 healthy controls. Plasma miRNA expression levels were quantified using quantitative real-time polymerase chain reaction. Diagnostic performance of individual miRNAs was assessed using receiver operating characteristic (ROC) curve analysis. A multivariable logistic regression model integrating multiple miRNAs was constructed to evaluate combined diagnostic performance, with internal validation performed using five-fold cross-validation. **Results**: Circulating miRNA expression profiles differed between TN patients and controls. Among individual markers, hsa-miR-183-5p demonstrated the highest diagnostic accuracy (AUC = 0.72), followed by hsa-miR-23a-3p (AUC = 0.65). hsa-miR-223-3p showed reversed directionality (AUC = 0.28), consistent with lower expression in TN and high specificity but low sensitivity at the optimal threshold. The combined miRNA panel achieved an apparent AUC of 0.86, with a mean cross-validated AUC of 0.84 ± 0.12, suggesting improved discrimination over single miRNAs but with variability consistent with the limited sample size. **Conclusions**: Apoptosis-related circulating miRNAs exhibit distinct expression patterns in idiopathic TN. While individual miRNAs show modest diagnostic performance, integration into a multi-miRNA panel improved discrimination between TN patients and healthy controls in this pilot dataset. These findings support the potential of apoptosis-based miRNA signatures as candidate minimally invasive biomarkers for TN, warranting further validation in larger, independent cohorts, ideally including clinically relevant disease-control facial pain conditions.

## 1. Introduction

Trigeminal neuralgia (TN) is a severe neuropathic pain disorder characterized by recurrent, short-lasting paroxysms of unilateral facial pain that are often described as electric shock–like and can be precipitated by innocuous triggers such as talking, chewing, or light touch. The condition imposes a substantial functional burden and can markedly reduce quality of life. Despite a well-defined clinical phenotype, the biological mechanisms underlying idiopathic trigeminal neuralgia (ITN) remain incompletely understood, and diagnosis continues to rely predominantly on clinical criteria rather than objective molecular measures [1,2]. Neuroimaging primarily serves to exclude secondary causes and to support etiologic classification, yet in routine practice the diagnostic task frequently involves differentiating TN from other facial pain syndromes with overlapping symptoms. This gap highlights the need for biologically informed, minimally invasive molecular markers that could complement clinical assessment and help refine mechanistic phenotyping in translational research.

Historically, TN has been strongly linked to focal demyelination and structural alterations within the trigeminal system, often discussed in relation to neurovascular compression and downstream electrophysiological instability [1]. However, structural explanations alone do not fully capture the heterogeneity of symptom course, chronicity, and variable therapeutic response observed across patients. Increasing evidence from the broader neuropathic pain literature supports a more integrative view in which neuroimmune–neuronal interactions contribute to the initiation and maintenance of pathological pain states. Neuroinflammation and neuroimmune activation—mediated by glial and immune cells and their downstream cytokine and chemokine signaling—can amplify nociceptive processing by altering neuronal excitability and synaptic transmission [3,4]. In parallel, activity-dependent plasticity within pain-processing circuits can increase the “gain” of nociceptive signaling, facilitating persistence and recurrence of symptoms even when the initial trigger is no longer prominent [5,6]. Within the trigeminal system, experimental work has further suggested that TN-like states may be accompanied by degenerative alterations and activation of apoptosis-related signaling pathways (e.g., CD95/CD95L), raising the possibility that pathways governing cellular stress responses, survival, and programmed cell death contribute to disease biology beyond purely mechanical hypotheses [7,8].

In this context, the inflammation–apoptosis axis has emerged as a plausible mechanistic framework for ITN. Apoptosis is essential for normal nervous system homeostasis, yet aberrant activation under pathological stress can compromise neuronal and glial integrity and may interact bidirectionally with inflammatory signaling [8,9]. Pro-inflammatory microenvironments can activate apoptotic cascades, while apoptosis-related structural and functional alterations may further promote inflammatory remodeling, creating a self-reinforcing loop that could plausibly sustain neuronal vulnerability and pain chronicity [3,7,8,9]. Molecular markers that reflect these intersecting processes are therefore of interest not only for mechanistic insight but also as candidate biomarkers to support objective stratification in clinical research.

MicroRNAs (miRNAs) are small non-coding RNAs that regulate gene expression post-transcriptionally and have broad roles in immune signaling, neuronal function, and stress responses [10,11,12]. Their relative stability in peripheral biofluids and their capacity to integrate upstream inflammatory and apoptotic signals make circulating miRNAs attractive candidates for minimally invasive biomarker discovery in neuropathic pain conditions [13,14]. However, miRNA research in TN remains limited, and existing studies vary substantially in candidate selection, analytical pipelines, and statistical modeling, which complicates cross-study comparability and the interpretation of diagnostic potential [13,14,15]. A small number of blood-based investigations have nonetheless supported the feasibility of detecting TN-associated circulating miRNA differences, motivating further exploration using explicitly defined biological hypotheses [15].

To address the heterogeneity of candidate discovery and to improve interpretability, we adopted a biologically targeted, a priori panel strategy focused on miRNAs implicated in neuroinflammation–apoptosis interactions and peripheral nerve biology. Specifically, we pre-specified six circulating miRNAs that represent complementary nodes within this mechanistic space while limiting multiple testing: miR-223 (linked to regulation of inflammatory signaling and neuroprotection) [16,17]; miR-23a (implicated in neuropathic pain–related inflammatory pathways and apoptosis-associated signaling) [18,19]; miR-186 (a stress-responsive regulator with context-dependent roles in apoptosis and cell survival) [20,21,22,23]; miR-183 (associated with neuronal vulnerability and apoptosis-related regulation) [24,25,26,27]; miR-222 (connected to Schwann cell behavior and myelination/remyelination-related processes) [28,29,30]; and miR-192 (a p53-responsive modulator of cellular stress responses) [31,32,33]. By focusing on a pre-defined, mechanism-informed panel rather than an unrestricted screening approach, we aimed to generate findings that are both biologically plausible and methodologically transparent as an initial exploratory step.

Accordingly, the primary objective of this study was to determine whether the circulating expression levels of these six miRNAs differ between patients with ITN and healthy controls, and to explore their ability to discriminate between groups using receiver operating characteristic (ROC) analyses. Recognizing that complex neuropathic pain biology is unlikely to be captured by any single molecular marker, we also evaluated whether combining multiple miRNAs in a multivariable model improves discrimination relative to individual miRNAs. We hypothesized that ITN would be associated with a distinct circulating miRNA pattern consistent with dysregulation in neuroimmune and apoptosis-related pathways, and that a multi-miRNA panel would provide improved classification performance compared with single-miRNA metrics. Importantly, given that our comparator group comprised healthy volunteers, the present work is intended as an exploratory signal-finding analysis that can inform subsequent, clinically oriented validation studies incorporating disease-control facial pain cohorts and independent external validation.

## 2. Materials and Methods

### 2.1. Study Design and Ethical Approval

This study was conducted at Afyonkarahisar Health Sciences University, Departments of Neurology and Medical Genetics, between 2025 and 2026 as a prospective case–control study. The study protocol was approved by the Afyonkarahisar Health Sciences University Medical Sciences Ethics Committee (Protocol Code: 2025/5; Approval Date: 11 April 2025) and was performed in accordance with the Declaration of Helsinki. Written informed consent was obtained from all participants prior to enrollment. No formal a priori power calculation was performed; the sample size was determined by feasibility and the study was designed as an exploratory/pilot case–control analysis.

### 2.2. Study Population

#### 2.2.1. Patient Group (Idiopathic Trigeminal Neuralgia)

The patient group consisted of 30 individuals diagnosed with idiopathic trigeminal neuralgia (TN). Patients were recruited from the Neurology outpatient clinic and evaluated according to the International Classification of Headache Disorders, 3rd edition (ICHD-3) [2] criteria. Inclusion criteria were:

- Age ≥ 18 years;
- Recurrent paroxysmal facial pain attacks affecting one or more branches of the trigeminal nerve;
- Short-lasting, severe, electric shock–like pain characteristics;
- Absence of neurological deficits on examination;
- Exclusion of secondary causes by magnetic resonance imaging (MRI), supporting an idiopathic TN diagnosis.

#### 2.2.2. Control Group

The control group included 20 healthy individuals evaluated under outpatient clinic conditions. Controls had:

- No diagnosis of trigeminal neuralgia;
- No chronic inflammatory or systemic disease;
- No regular medication use related to systemic inflammation or chronic illness.

All controls were clinically assessed and included after confirmation of eligibility and written informed consent.

### 2.3. Exclusion Criteria

Participants were excluded if they had:

- Secondary trigeminal neuralgia due to tumors, intracranial lesions, multiple sclerosis, herpes zoster, or neurovascular compression;
- Cognitive or psychiatric disorders impairing participation;
- Severe hepatic or renal insufficiency;
- Known coagulation disorders;
- Diagnosed malignancy or ongoing cancer treatment;
- Chronic inflammatory diseases (e.g., rheumatoid arthritis, systemic lupus erythematosus, inflammatory bowel disease);
- Regular use of immunosuppressive or anti-inflammatory medications.

### 2.4. Collection of Peripheral Blood

From the patients who applied to the Afyonkarahisar Health Sciences University, Facuty of Medicine, Neurology Polyclinic, 10 mL of venous blood samples were taken from the forearm with a vacutainer system into tubes containing ethylenediamine tetraacetic acid, and then centrifuged at 3600 rpm (2800× *g*) for 10 min within 30 min of collection. Subsequently, a total of 4 mL of plasma was dispensed into two separate 2 mL Eppendorf tubes for each sample and stored at −80 °C for later use.

### 2.5. Sample Collection and miRNA Isolation

Total RNAs were isolated from individual 200 μL frozen plasmas using the miRNeasy Serum/Plasma Advanced Kit (cat. no. 217204; Qiagen, Dusseldorf, Germany) according to the manufacturer’s instructions. The isolated RNAs were stored for 1 year at −80 °C without visible degradation. The concentration and purity of the RNA samples obtained were determined by measuring their absorbance at 260/280 nm wavelengths. Measurements were performed on a Nanodrop (Thermo Scientific, Waltham, MA, USA) device using 1–1.5 μL of each sample. The purity of the isolated RNAs was checked by the ratio of their absorbance at 260 nm and 280 nm.

### 2.6. Reverse Transcription-Quantitative Polymerase Chain Reaction

The reverse transcription step was performed with the miRCURY LNA RT Kit (cat. no. 339340; Qiagen) using 2 μL of each total RNA (10 ng/mL) according to the kit protocol manual, with a total volume of 10 µL. 5S rRNA (cat.no. YP00203906) was used as reference genes. Reference genes used for normalization should exhibit stable expression across all samples, be essential for cellular viability, and remain unaffected by cellular or experimental conditions. In this study, the UniSP6 spike-in control provided in the miRCURY LNA Kit was employed for normalization. Reverse transcription was performed at 42 °C for 60 min, followed by enzyme inactivation at 95 °C for 5 min. The reaction contained primers for hsa-miR-183-5p (YP00206030), hsa-miR-23a-3p (YP00204722), hsa-miR-223-3p (YP00205986), hsa-miR-222-5p (YP00204314), hsa-miR-192-5p (YP00204099), and hsa-miR-186-5p (YP00206053) and 5S rRNA (cat.no. YP00203906). For each quantitative polymerase chain reaction (qPCR), 3 μL of cDNA generated using the miRCURY LNA RT Kit was used as template. Amplification reactions were carried out with the miRCURY LNA SYBR Green PCR Kit (cat. no. 339346) on a Rotor-GeneQ real-time PCR system (Qiagen). The cycling protocol consisted of an initial denaturation step at 95 °C for 2 min, followed by 50 amplification cycles of 10 s at 95 °C and 60 s at 56 °C, in accordance with the manufacturer’s recommendations. cDNA synthesis quality was assessed using 5S rRNA. Relative miRNA expression levels were determined using the comparative threshold cycle (Ct) approach, with 5S rRNA serving as the reference gene for normalization. Transcript levels were reported as fold change relative to the healthy control group, calculated using the 2^−ΔΔCt^ method.

### 2.7. Statistical Methods and Software

Statistical analyses were performed to investigate the diagnostic value and potential biological relevance of the selected miRNAs in trigeminal neuralgia (TN). All analyses were conducted using established statistical software. Preliminary data processing, normalization, and exploratory assessment of miRNA expression profiles were carried out using the QIAGEN GeneGlobe Data Analysis Center according to the manufacturer’s protocol. Receiver operating characteristic (ROC) curve analyses, including the calculation of area under the curve (AUC), sensitivity, and specificity, were performed using IBM SPSS Statistics version 20.0 (IBM Corp., Armonk, NY, USA).

ROC curve analysis was used to evaluate the discriminatory ability of individual miRNAs to differentiate TN patients from healthy controls. The AUC was used as a quantitative measure of diagnostic performance. AUC values were interpreted according to conventional thresholds: 0.5–0.6 indicating no discrimination, 0.6–0.7 poor discrimination, 0.7–0.8 acceptable discrimination, 0.8–0.9 excellent discrimination, and values above 0.9 representing outstanding discriminatory performance.

To assess the combined diagnostic potential of multiple miRNAs, a multivariable logistic regression model incorporating all candidate miRNAs was constructed. A combined ROC curve was subsequently generated using predicted probabilities derived from the regression model. The statistical significance of ROC curves and differences between observed AUC values and random classification were evaluated using the DeLong test.

Optimal diagnostic thresholds for each miRNA were determined using the Youden index (J = sensitivity + specificity − 1), which identifies the point that maximizes the joint performance of sensitivity and specificity.

Principal component analysis (PCA) was performed to explore overall variance structure and potential clustering patterns between TN patients and controls based on normalized miRNA expression data.

Volcano plot analysis was applied to illustrate differential miRNA expression by integrating fold-change values (Ct-based differences) with statistical significance, thereby enabling identification of miRNAs showing both biologically meaningful and statistically significant alterations.

To examine the relative contribution of each miRNA within the multivariable model, logistic regression coefficients were evaluated as indicators of feature importance. Model robustness and generalizability were further assessed using five-fold cross-validation (5-fold CV), and the mean AUC together with the corresponding standard deviation across folds was calculated to estimate model stability.

A *p*-value < 0.05 was considered statistically significant for all analyses.

Descriptive statistics were expressed as mean ± standard deviation or median (minimum–maximum), as appropriate. Normality was assessed prior to group comparisons. Between-group comparisons were conducted using the independent samples *t*-test for normally distributed variables, the Mann–Whitney *U* test for non-normally distributed variables, and Fisher’s exact test for categorical variables.

## 3. Results

### 3.1. Study Population and Baseline Characteristics

A total of 50 participants were included in the study, comprising 30 patients with idiopathic trigeminal neuralgia (TN) and 20 healthy controls. The two groups were comparable in terms of age (54.4 ± 12.9 vs. 52.8 ± 12.0 years; *p* = 0.645), body mass index (27.5 ± 3.8 vs. 26.7 ± 1.5 kg/m^2^; *p* = 0.452), sex distribution (female: 76.7% vs. 80.0%; *p* = 1.000), smoking status (*p* = 0.548), and alcohol use (none in either group). Although comorbid conditions were more frequent in the TN group, this difference did not reach statistical significance (*p* = 0.067) (Table 1).

Within the TN cohort, the mean age was 54.4 ± 12.9 years (range: 30–81). The median disease duration was 8 years (range: 1–30). The mean number of daily pain attacks was 10.8 ± 7.7, and the mean pain intensity assessed by the Visual Analog Scale (VAS) was 8.24 ± 0.88. Pain localization most commonly involved the V3 branch (40%), followed by V2 (20%), V1 + V2 (16.7%), V1 + V2 + V3 (20%), and isolated V1 involvement (3.3%). Right-sided involvement was observed in 73.3% of patients (Table 2).

### 3.2. Circulating miRNA Expression Profiles

**Figure 1:**
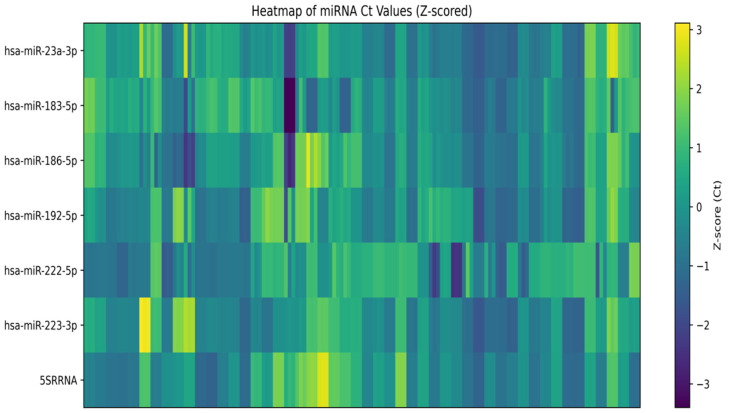
Heatmap of z-score–normalized circulating miRNA Ct values in idiopathic trigeminal neuralgia and healthy controls.

**Figure 2:**
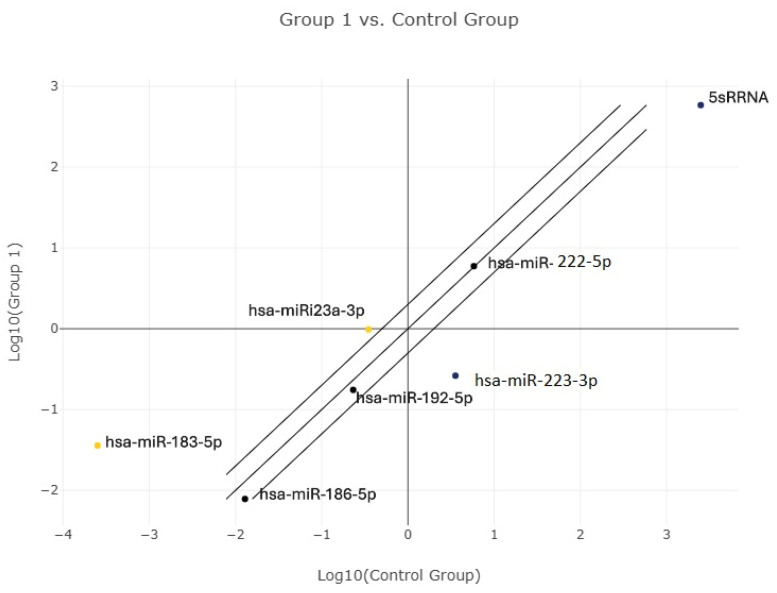
GeneGlobe scatter plot illustrating relative expression of apoptosis-related miRNAs in idiopathic trigeminal neuralgia patients versus healthy controls.

Differential expression analysis of circulating plasma miRNAs was initially performed using normalized expression values derived from ΔΔCt calculations in the QIAGEN GeneGlobe Data Analysis Center. Group comparisons were conducted using normalized Ct values, and relative fold-change trends between TN patients and healthy controls were explored prior to ROC-based diagnostic evaluation. Analysis of circulating apoptosis-related miRNAs demonstrated distinct expression patterns between TN patients and healthy controls. Heatmap visualization based on z-score–normalized Ct values revealed differential clustering of samples according to disease status (Figure 1). In particular, hsa-miR-183-5p and hsa-miR-23a-3p clustered together and exhibited relatively homogeneous expression patterns in the TN group, whereas hsa-miR-223-3p showed consistent down-regulation and separated from other miRNAs. GeneGlobe scatter plot analysis further illustrated the relative expression distribution of apoptosis-related miRNAs between TN patients and healthy controls, supporting the distinct expression patterns observed in the heatmap (Figure 2).

Among the analyzed miRNAs, miR-223-3p demonstrated the strongest biomarker potential, showing the largest expression difference between TN patients and controls (fold change = 1.27, 95% CI: 1.22–1.29, *p* < 0.001). Significant down-regulation was also observed for miR-183-5p and miR-23a-3p, although with smaller effect sizes. No significant differences were detected for miR-186-5p, miR-192-5p, or miR-222-5p (Table 3).

### 3.3. Diagnostic Performance of Individual miRNAs

Receiver operating characteristic (ROC) curve analysis was performed using normalized miRNA expression values to evaluate the discriminative ability of six circulating miRNAs between TN patients and healthy controls.

- hsa-miR-183-5p demonstrated the highest individual diagnostic performance, with an AUC of 0.72 (*p* < 0.001), a Youden cut-off value of 33.20, sensitivity of 75.6%, and specificity of 70%.
- hsa-miR-23a-3p showed moderate discriminative ability (AUC = 0.65, *p* = 0.0026), with a Youden cut-off of 24.35, sensitivity of 54%, and specificity of 85%.
- hsa-miR-192-5p exhibited limited discriminative power (AUC = 0.55, *p* = 0.27).
- hsa-miR-222-5p also showed low diagnostic accuracy (AUC = 0.54, *p* = 0.42).
- hsa-miR-186-5p did not demonstrate meaningful discriminative performance (AUC = 0.49, *p* = 0.78).
- hsa-miR-223-3p demonstrated reversed directionality (AUC = 0.28, *p* < 0.001), consistent with lower expression in TN patients. At the selected threshold, specificity was high (100%) but sensitivity was low, indicating limited standalone diagnostic utility.

Detailed diagnostic metrics for all analyzed miRNAs are presented in Table 4.

### 3.4. Combined miRNA Panel Analysis

**Figure 3:**
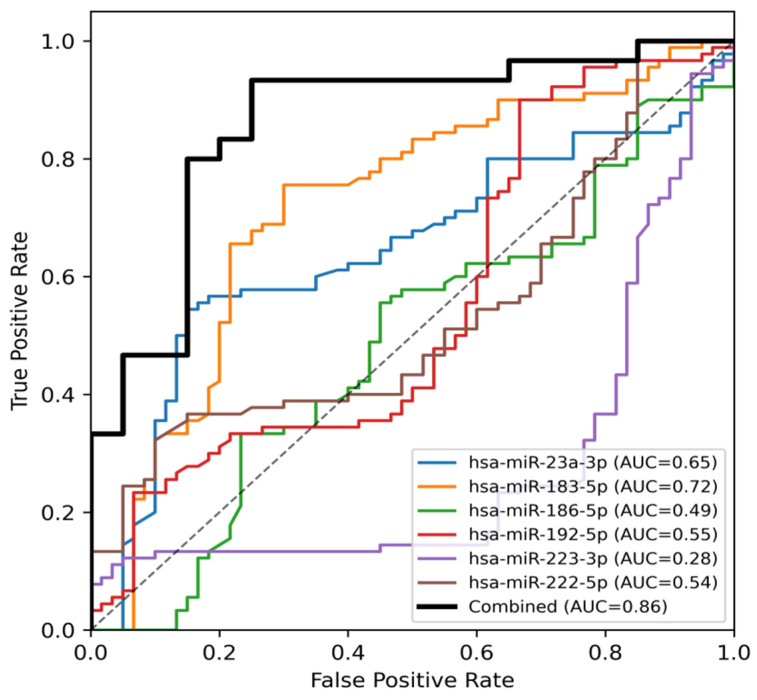
Receiver operating characteristic (ROC) curves of individual apoptosis-related miRNAs and the combined miRNA panel in idiopathic trigeminal neuralgia. The dashed line represents the line of no discrimination (AUC = 0.5).

To evaluate the collective diagnostic contribution of multiple miRNAs, a multivariable logistic regression model incorporating all apoptosis-related miRNAs was constructed. The combined miRNA panel demonstrated high overall diagnostic performance, with an AUC of 0.86 on ROC analysis (Figure 3), exceeding the performance of all individual miRNA markers.

In internal validation analysis, the combined miRNA panel achieved a mean cross-validated AUC of 0.84 ± 0.12, which was only slightly lower than the apparent AUC of 0.86 obtained using the full dataset. This finding suggests a degree of model stability; however, given the limited sample size, potential optimism bias cannot be fully excluded.

Hierarchical clustering and combined ROC analyses consistently demonstrated that integration of multiple miRNAs provided superior discrimination between TN patients and healthy controls compared with single-marker evaluations (Figure 3).

## 4. Discussion

Trigeminal neuralgia (TN) is a neuropathic pain syndrome characterized by severe and recurrent paroxysmal pain attacks, the etiology of which has not yet been fully elucidated. Recent studies have demonstrated that chronic irritation of the trigeminal nerve, demyelination, inflammation, and accompanying apoptotic processes play important roles in the pathophysiology of the disease [1,3,4,6,7]. In this context, microRNAs (miRNAs), which regulate gene expression at the post-transcriptional level, have attracted increasing attention both for understanding the molecular mechanisms of TN and for the development of diagnostic biomarkers [11,13,14].

In the present study, expression profiles of selected inflammation- and apoptosis-related miRNAs were evaluated in patients with TN compared with healthy controls. A comprehensive analytical approach was adopted, incorporating ROC analysis, AUC ranking, z-score–normalized heatmaps, and hierarchical clustering. Our findings indicate that, beyond single-miRNA assessments, models integrating multiple miRNAs may better capture the heterogeneous biological nature of TN.

The clinical severity observed in our cohort (mean VAS 8.24; daily attacks 10.8) is consistent with previously reported TN cohorts, where VAS scores typically range between 7 and 9 and attack frequency varies according to disease duration and treatment status [1,15]. Thus, our cohort represents a clinically active TN population.

The main findings of this study can be summarized under three key points: (i) hsa-miR-183-5p emerged as a promising biomarker signal in this exploratory cohort, exhibiting the highest diagnostic performance (highest AUC in ROC analysis) together with a consistent expression pattern in the patient group; (ii) hsa-miR-23a-3p, which showed the second-highest AUC, contributed to discriminatory performance through its stable and directionally consistent regulation, independent of fold-change magnitude; and (iii) under conditions where individual biomarkers displayed limited to moderate performance, the combined miRNA panel showed higher apparent discrimination, although these findings should be interpreted cautiously given the sample size and the multivariable nature of the model.

The miR-183 family has been extensively characterized in the literature as a key regulator of neuronal survival, mitochondrial function, and apoptotic signaling pathways [24,26,27]. Experimental studies have demonstrated that miR-183-5p modulates neuronal fate through its effects on caspase activation, BCL-2 family proteins, and mitochondrial apoptosis cascades [24,25]. Given that chronic nerve injury and sustained neuronal stress in trigeminal neuralgia are known to trigger apoptotic mechanisms [1,7,8], dysregulation of miR-183-5p represents a biologically plausible contributor to disease pathophysiology. In line with this biological background, our study identified hsa-miR-183-5p as the most prominent signal in this dataset, demonstrating the highest AUC value in ROC analysis and a highly consistent expression pattern across both heatmap and GeneGlobe clustering analyses. Taken together, the convergence of robust biological evidence and strong diagnostic performance supports further investigation of hsa-miR-183-5p as a circulating biomarker candidate, while acknowledging that the observed AUC is modest and requires independent validation.

In the literature, miR-23a-3p is well recognized as an anti-apoptotic microRNA involved in the suppression of BIM (BCL2L11), FAS/FASL signaling, and caspase-mediated apoptotic pathways. Experimental studies have demonstrated that increased neuronal expression of miR-23a-3p promotes cell survival by limiting apoptosis following neural injury [18,19,34,35]. In the context of trigeminal neuralgia, this regulatory profile suggests a possible compensatory or homeostatic role aimed at counterbalancing chronic injury-induced apoptotic stress [1,7,8]. In line with this biological background, hsa-miR-23a-3p exhibited the second-highest AUC value in ROC analysis in our study. Although GeneGlobe scatter analysis did not reveal a pronounced fold-change, the miRNA demonstrated stable and directionally consistent regulation across individuals, which likely underlies its discriminatory capacity. This observation underscores that, in biomarker evaluation, consistency of expression may be as critical as the magnitude of expression change. Accordingly, rather than serving as a strong standalone biomarker, hsa-miR-23a-3p appears to function as a supportive or stabilizing component that may enhance diagnostic robustness when incorporated into combined biomarker panels, but its modest AUC indicates that clinical utility remains exploratory and requires validation.

hsa-miR-222-5p, a member of the miR-221/222 family, is associated with regulation of cell proliferation, cellular adaptation, and stress responses. Experimental studies have demonstrated that this miRNA family can modulate proliferation and cellular behavior in peripheral nervous system cells [28,29]. Moreover, miR-221/222 family members have been implicated in inflammatory responses, cellular stress, and apoptosis-related pathways in the neuropathic pain and inflammation literature [29,30]. In the present study, hsa-miR-222-5p showed reduced expression in TN patients compared with controls; however, ROC analysis did not reveal statistically significant discriminative performance, indicating limited diagnostic utility. Heatmap and clustering analyses further demonstrated marked inter-individual variability in expression. Taken together, these findings suggest that hsa-miR-222-5p is not a strong standalone diagnostic biomarker for TN, but may function as a secondary molecular indicator reflecting changes in stress- and survival-related pathways. The available data support the interpretation that the role of miR-222-5p is context- and cell-type dependent, and that in neuropathic pain conditions it may primarily reflect accompanying biological processes rather than disease presence per se.

hsa-miR-223-3p is among the miRNAs involved in regulating the interaction between inflammation and apoptosis, particularly through modulation of immune cell activity and inflammatory responses. Previous studies have shown that miR-223-3p can suppress apoptotic processes via caspase-3 activity and inflammasome-related pathways [16,17]. In this study, hsa-miR-223-3p exhibited markedly reduced expression in TN patients compared with controls. An AUC value of 0.28 in ROC analysis indicates reversed directionality rather than a true “inverse” diagnostic effect; when directionality is inverted, this corresponds to an AUC of 0.72. Heatmap analysis further demonstrated consistently higher Ct values and uniformly low expression levels in TN patients. Based on these findings, hsa-miR-223-3p may be considered a negative diagnostic biomarker rather than a positive marker for TN. Its diagnostic contribution may be particularly relevant as a directionally informative marker within a combined panel; however, the very low sensitivity observed does not support an exclusion (“rule-out”) function.

hsa-miR-192-5p is primarily associated with p53-mediated cellular stress responses, DNA damage, and apoptotic processes. Experimental studies have shown that miR-192 is induced by p53 and contributes to cell-cycle arrest and suppression of proliferation [31,32]. In contrast, hsa-miR-186-5p has been implicated in regulation of apoptotic signaling under cellular stress and neurotoxic conditions, with context-dependent effects mediated through long non-coding RNA–miRNA interactions [20,21,22]. These observations highlight the context-specific regulatory roles of miRNAs across different tissues and pathological conditions. In the present study, both hsa-miR-192-5p and hsa-miR-186-5p demonstrated low AUC values and no marked expression differences between TN patients and controls. Heatmap and ROC analyses confirmed the absence of consistent and meaningful separation between groups for these miRNAs. Therefore, hsa-miR-192-5p and hsa-miR-186-5p should not be considered primary diagnostic biomarker candidates for TN, but rather secondary molecular indicators reflecting cellular stress and apoptotic processes accompanying the disease.

Although individual miRNAs exhibited limited to moderate diagnostic performance, the combined miRNA model showed higher apparent AUC values than individual markers. The high AUC value obtained from the multivariable miRNA panel suggests that the heterogeneous pathophysiology of TN cannot be adequately represented by a single molecular marker. Instead, simultaneous evaluation of multiple miRNAs related to inflammation, apoptosis, and cellular stress appears to provide a more biologically and diagnostically realistic approach. This finding establishes a strong conceptual foundation for the future development of multi-biomarker panels in TN, while recognizing the need for external validation and caution regarding potential overfitting in small cohorts.

### 4.1. General Evaluation and Clinical Implications

Overall, our findings indicate that apoptosis-related miRNA dysregulation constitutes an important component of the molecular pathophysiology of TN. In particular, the prominence of hsa-miR-183-5p and hsa-miR-23a-3p in terms of both biological relevance and diagnostic performance suggests that their combined evaluation may enhance discrimination between TN patients and healthy individuals. This supports the notion that multi-miRNA panels may provide more reliable and clinically meaningful results in scenarios where individual miRNAs show limited discriminative power, but the present results should be considered exploratory.

From a clinical perspective, apoptosis-related miRNA profiles may reflect underlying cellular stress and neuronal injury processes in TN. The ability to assess these miRNAs using minimally invasive plasma-based methods raises the possibility of their future use as supportive diagnostic tools or for monitoring disease biology. Nevertheless, translation into clinical practice will require larger, prospective, and multicenter validation studies, including clinically relevant disease-control cohorts.

### 4.2. Study Limitations

This study has several limitations. It was conducted as a single-center case–control study with a relatively small sample size, which may limit generalizability. Moreover, comparison with healthy volunteers rather than clinically relevant disease-control cohorts (e.g., other facial pain or neuropathic pain conditions) may introduce spectrum bias and inflate apparent diagnostic performance. Future studies including appropriate disease-control groups are necessary to assess real-world discriminative validity.

The cross-sectional design captures only a single time point and does not allow causal inference. In addition, plasma-based miRNA measurements may not directly reflect local trigeminal tissue biology; thus, mechanistic interpretations should be considered literature-based hypotheses. Clinical heterogeneity could not be explored through subgroup analyses due to sample size constraints, and external validation in larger, independent cohorts remains essential.

Finally, the use of 5S rRNA as an endogenous reference gene may represent a methodological limitation, as the stability of small nuclear RNAs in circulating biofluids is debated. Alternative normalization strategies should be considered in future validation studies.

## 5. Conclusions

In conclusion, this study demonstrates that selected apoptosis-related circulating plasma miRNAs exhibit distinct expression profiles in patients with trigeminal neuralgia. The findings suggest that miRNA dysregulation related to apoptotic pathways may represent a meaningful component of TN molecular pathophysiology. Among the analyzed miRNAs, hsa-miR-183-5p emerged as the most promising signal in this exploratory cohort based on its diagnostic performance and consistent expression pattern, while hsa-miR-23a-3p provided modest supportive discriminatory power within combined biomarker panels. Conversely, hsa-miR-223-3p showed down-regulation in TN and an AUC below 0.5, indicating reversed directionality rather than a clinically applicable “exclusion” marker; its potential utility requires further validation.

When individual miRNAs exhibit limited diagnostic performance, multi-miRNA panels may increase apparent discrimination, but these multivariable findings should be interpreted cautiously due to the small sample size and require confirmation in independent cohorts, ideally including clinically relevant disease-control groups. Collectively, these results indicate that apoptosis-based miRNA profiles may contribute both to understanding the biological basis of TN and to the future exploration of biomarker-based diagnostic approaches.

## Figures and Tables

**Table 1:** Comparison of demographic characteristics, lifestyle factors, and comorbidities between patient and control groups.

Variable	Patients (*n* = 30)	Controls (*n* = 20)	Test Statistic	*p*-Value
Age (years) †	54.4 ± 12.9 (Median: 55)	52.8 ± 12.0 (Median: 51)	t = 0.46	0.645
BMI (kg/m^2^) ‡	27.53 ± 3.78 (Median: 27.2)	26.74 ± 1.46 (Median: 27.1)	U = 338.5	0.452
Sex §	Female: 23/Male: 7	Female: 16/Male: 4	-	1.000
Smoking status §	No: 21/Yes: 9	No: 12/Yes: 8	-	0.548
Alcohol consumption ¶	No: 30/Yes: 0	No: 20/Yes: 0	-	-
Presence of comorbidities §	No: 22/Yes: 8	No: 19/Yes: 1	-	0.067

Abbreviations: BMI, body mass index; Statistical tests: † Independent samples *t*-test; ‡ Mann–Whitney U test; § Fisher’s exact test; ¶ Test not applicable due to constant variable.

**Table 2:** Clinical characteristics of patients with trigeminal neuralgia (TN).

Patient Characteristics	TN (Total)
Number of patients	30
Sex (female/male), *n*	23/7
Age (years), mean ± SD (range)	54.4 ± 12.9 (30–81)
Disease duration—TN (years), median (range)	8 (1–30)
Daily attack frequency (attacks/day), mean ± SD (range)	10.8 ± 7.7 (1–30)
Pain intensity—VAS (0–10), mean ± SD (range)	8.24 ± 0.88 (6–9)
Pain localization, % (V1/V2/V3/multiple branches)	V1: 3.3%; V2: 20%; V3: 40%; V1 + V2: 16.7%; V1 + V2 + V3: 20%
Laterality, n (%)	Right: 22 (73.3%); Left: 6 (20.0%); Bilateral tendency (Right > Left): 2 (6.7%)
Medications used prior to enrollment, *n* (%)	
Carbamazepine (CBZ)	11 (36.7%)
Pregabalin + CBZ combination	3 (10.0%)
Other/multiple regimens (e.g., CBZ + duloxetine)	3 (10.0%)
No medication	13 (43.3%)
Family history of TN, n (%)	0 (0%)
History of surgical or procedural intervention, n (%)	
No previous surgery	27 (90.0%)
Radiofrequency (RF) ablation	2 (6.7%)
Non-surgical procedural treatment	1 (3.3%)

Abbreviations: TN, trigeminal neuralgia; SD, standard deviation; VAS, visual analog scale; V1, ophthalmic branch; V2, maxillary branch; V3, mandibular branch; CBZ, carbamazepine; RF, radiofrequency.

**Table 3:** Expression levels of analyzed miRNAs in TN patients and controls. Values are presented as median (IQR). Between-group comparisons were performed using the Mann–Whitney U test.

miRNA	TN Median (IQR)	Control Median (IQR)	Fold Change	95% CI	*p*-Value
hsa-miR-23a-3p	23.74 (5.70)	26.30 (3.43)	0.90	0.87–0.98	0.0026
hsa-miR-183-5p	30.54 (4.72)	34.72 (3.80)	0.88	0.85–0.92	<0.001
hsa-miR-186-5p	27.98 (5.75)	28.52 (4.01)	0.98	0.95–1.03	0.775
hsa-miR-192-5p	27.92 (5.28)	26.74 (7.28)	1.04	0.92–1.11	0.266
hsa-miR-222-5p	22.05 (5.00)	21.36 (3.10)	1.03	0.96–1.09	0.422
hsa-miR-223-3p	32.97 (5.02)	25.95 (3.37)	1.27	1.22–1.29	<0.001
5SRRNA	16.84 (2.68)	16.55 (3.12)	1.02	0.96–1.09	0.440

**Table 4:** Diagnostic performance of circulating miRNAs in trigeminal neuralgia.

miRNA	AUC	*p* Value	Youden Ct Cut-off	Sensitivity	Specificity
hsa-miR-183-5p	0.72	<0.001	33.20	0.76	0.70
hsa-miR-23a-3p	0.65	0.0026	24.35	0.54	0.85
hsa-miR-192-5p	0.55	0.27	31.79	0.90	0.33
hsa-miR-222-5p	0.54	0.42	19.99	0.32	0.90
hsa-miR-186-5p	0.49	0.78	28.20	0.56	0.55
hsa-miR-223-3p	0.28	<0.001	22.20	0.08	1.00

## Data Availability

The data presented in this study are available from the corresponding author upon reasonable request.
